# Causes of Reduced Vision in Australian Children

**DOI:** 10.1007/s44402-026-00128-x

**Published:** 2026-06-18

**Authors:** Mythili Ilango, Kathryn Ailsa Rose, Felicia Christabelle Adinanto, Amanda Nicole French

**Affiliations:** University of Technology Sydney, Sydney, New South Wales Australia

**Keywords:** Myopia, Paediatric, Reduced vision, Uncorrected refractive error, Vision screening

## Abstract

**Purpose:**

To determine the prevalence and causes of reduced vision in childhood.

**Methods:**

Cross-sectional analysis included five cohorts aged; 3–5 years old (*n* = 986) from the Sydney Paediatric Eye Disease Study (SPEDS), 6 years (*n* = 1739) and 12 years (*n* = 2345) from the Sydney Myopia Study (SMS) and the 5 year follow-up of SMS at 12 years (*n* = 1111) and 17 years (*n* = 1649) in the Sydney Adolescent and Vascular Eye disease Study (SAVES). All children had a comprehensive ocular examination, including visual acuity (VA) with and without refractive correction. Reduced uncorrected VA was defined as worse than 6/12 (logMAR 0.30). A longitudinal analysis was also conducted to identify incident cases of reduced vision between SMS baseline and SAVES follow-up among children assessed at both time points.

**Results:**

The overall prevalence of reduced vision based on unaided visual acuity increased with age from 4.5% in SPEDS 3–5 year olds to 17.7% in SAVES 17 year olds (*p* < 0.001). The proportion of reduced vision caused by refractive error, amblyopia, strabismus and pathology also varied between cohorts. In the younger cohorts, amblyopia accounted for 21% of SPEDS 3–5 year olds and 28% of SMS 6 year olds with reduced vision. There was a substantial decline in the proportion of reduced vision attributed to amblyopia in the older cohorts, while refractive error increased. Myopia contributed to 18% of children with reduced vision in SPEDS 3–5 years and SMS 6-year-olds and increased to over 80% in SMS 12-year-olds, SAVES 12-year-olds and 17-year-olds.

**Conclusions:**

Targeted preschool screening for amblyopia is supported as it occupied a higher proportion of the younger ages. Community education to promote wearing glasses in adolescence for those with myopia is warranted.

Key Points

- Preschool vision screening remains the most effective approach for identifying conditions requiring early treatment, particularly amblyopia, supporting timely intervention and improved long-term visual outcomes.
- The eye conditions associated with reduced vision differ across childhood, suggesting that screening and eye care approaches should reflect changing visual health needs as children grow.
- Population-wide repeated vision screening during primary school may offer limited benefit, while targeted detection of myopia and improved eye health awareness in older children may be more effective.

## Introduction

Vision impairment is ranked 6th according to the global burden of disease [1]. At least 2.2 billion people worldwide have a vision impairment and of these, at least 1 billion have an ocular condition that could have been prevented or treated [2]. Although the majority of those with vision impairment are over the age of 50 years, childhood vision impairment accounts for 4% of global visual impairment, yet one third of the costs associated with vision impairment and blindness is attributed to children’s vision impairment [3]. A recent study found that uncorrected refractive error was one of the leading causes of blindness and moderate to severe vision impairment in children, with the authors recommending interventions at schools to improve effective coverage for those who require spectacles [4].

A number of childhood ocular conditions, including refractive error and amblyopia, are treatable. However, particularly for amblyopia, treatment is time sensitive to the period of neural plasticity, and early treatment has been shown to be most effective for reversing visual impairment [4–8]. There is also a potential benefit of timely treatment to minimise the impact of reduced vision on a child’s ability to learn, socialise and participate in daily activities, as well as the costs associated with vision impairment continuing into adulthood [3, 4]. A focus group study conducted for 6–12-year-old children with vision impairment found a significant impact on quality of life. The most common area of concern was the ability to cope at school, including having access to appropriate print size, assistive devices and experiencing reading difficulties (21%). Also reported were future expectations and frustrations such as: lack of cure for the condition or worsening over time (14%) and psychosocial wellbeing (13%) [9]. In the circumstance that visual impairment cannot be treated, early detection remains valuable for the provision of low vision aids and support services [10–13]. Coupled with the need for early treatment, this adds a further argument for the importance of vision screening in childhood.

Current recommendations for vision screening indicate that a target age of 3–5 years is most appropriate for treatment of amblyopia and correction of refractive errors prior to school entry [14–17]. As the prevalence of eye conditions detected by vision screening, including amblyopia, strabismus and refractive error, differs with location, ethnicity and socioeconomic status among other population-specific factors, the referral rate from vision screening programmes can vary with the community being screened [4, 18–33]. The aim of this study is to determine the proportion of children with reduced vision in Sydney, Australia and the conditions likely to be detected by vision screening. An additional question related to vision screening is whether repeated screening at older ages may be necessary to detect new cases of reduced vision, particularly with the onset of myopia. As such, a further aim is to investigate the incidence of reduced vision in school-aged children enroled in a longitudinal cohort and assess the necessity for additional screening at older ages.

## Method

### Study Design and Participants

This study draws on data from three large, population-based studies of Australian children conducted in metropolitan Sydney, Australia. The Sydney Paediatric Eye Disease Study (SPEDS) cross-sectionally sampled children aged 6 months to 6 years from 2007 to 2009, with children aged 3–5 years included in the current analysis. The Sydney Myopia Study (SMS) sampled schoolchildren in two age cohorts at 6 and 12 years (2003–2005). Children who participated in SMS were followed up 5–6 years later as part of the Sydney Adolescent Vascular and Eye Study (SAVES) between 2009 and 2011, at ages 12 and 17 years. Children who were in the same school year during SAVES and who were not examined at baseline in SMS were additionally invited to participate and are included in cross-sectional analyses. A longitudinal analysis of new cases of reduced vision between baseline (SMS) and follow-up examination (SAVES) included only children assessed at both time points.

All studies used population-based sampling designs, with detailed recruitment strategies described previously [25, 34, 35]. In brief, random cluster sampling based on Australian Bureau of Statistics socioeconomic strata was employed to ensure a representative sample was obtained. In the SPEDS study of preschool-age children, eligible households within selected geographic areas were identified from census maps and recruited by door-knocking and mailouts. For the SMS study, 34 primary schools and 21 secondary schools were randomly selected from socioeconomic strata. A representative mix of private, public and religious schools was included. All children in the eligible school year (years 1 and 6) were invited to participate. The SAVES study returned to schools that participated in SMS for longitudinal follow-up of children, and all children in years 6–7 and years 11–12 were eligible to participate. No exclusion criteria were applied, with only children who did not consent or were unable to complete the assessment not included.

Ethics approval was obtained from the Human Research Ethics Committee of the University of Sydney. Additional approvals were obtained from the New South Wales Department of Education and the Catholic Education Office for SMS and SAVES. All studies adhered to the Declaration of Helsinki. Written informed consent was obtained from parents or guardians (or directly from participants aged ≥18 years), with verbal assent from participating children.

### Examination Procedures

All children underwent a comprehensive ocular examination conducted by orthoptists and medical officers with ophthalmology consultation, as required. Standardised and age-appropriate procedures were utilised across all studies.

Uncorrected (unaided) visual acuity (VA) and presenting VA (with habitual refractive correction, if present) were obtained for all children. Current spectacle prescriptions were measured using focimetry for children wearing spectacles. Children aged 3–5 years (SPEDS) were examined using the Electronic Visual Acuity HOTV system, using Amblyopia Treatment Study protocols [36]. For children aged 6, 12 and 17 years (SMS and SAVES), VA was measured using a 2.44 m retro-illuminated logMAR Early Treatment of Diabetic Retinopathy Study chart. Pinhole VA was measured in older children (SMS and SAVES) when VA was ≥0.10 logMAR or there was one line or more difference between the two eyes. Best-corrected visual acuity (BCVA) was measured for children between 12 and 17 years of age, following subjective refraction. Subjective refraction was not performed for younger children, aged 3–5 years (SPEDS) and 6 years (SMS).

Cycloplegic autorefraction was performed using Retinomax (Visionix, visionix.com) in younger children (SPEDS) and the Canon RK-F1 (Canon, us.medical.canon) for older children (SMS and SAVES). The cycloplegia protocol (all eye drops—Bausch + Lomb, minims range, bausch.com) included amethocaine 0.5%, followed by both cyclopentolate 1% and tropicamide 1% instilled in two cycles, 5 min apart. Additional phenylephrine 2.5% was included where dilation was inadequate, particularly in children with dark irides.

Orthoptic assessment of ocular alignment was determined by cover test and measured using a prism bar cover test at near and distance fixation. Ocular motility and the near point of convergence were also measured. Stereopsis was screened for all children using Lang II (Lang-Stereotest AG, lang-stereotest.com), with age-appropriate stereoacuity measured by Stereosmile (Vision Assessment Corporation, visionassessment.com) or Randot Preschool Test (Stereo Optical Company, stereooptical.com) for young children (<6 years)and TNO (Lameris Ootech BV, lameris-group.nl) in older children (≥6 years). Ocular pathology was identified using slit lamp biomicroscopy to examine anterior ocular structures and dilated posterior examination using indirect ophthalmoscopy for younger children (<6 years) and fundus photography in children aged 3 years and older.

### Definitions

Reduced vision was defined as uncorrected (unaided) VA worse than 0.30 logMAR (6/12) in either eye. Where reduced uncorrected VA was improved with the habitual refractive correction (presenting VA) or BCVA, or if uncorrected refractive error was present on autorefraction, then this was determined to be ‘correctable’ reduced vision. The severity of reduced vision was classified according to the World Health Organization definitions [2], i.e., mild <0.30 logMAR (<6/12), moderate <0.48 logMAR (<6/18) and severe <1.00 logMAR (<6/60). Bilateral reduced vision was defined as uncorrected VA in both eyes, with severity classified according to the better-seeing eye.

Unilateral amblyopia was defined as an interocular difference in VA of ≥two lines (0.20 logMAR or a 10-letter difference) and bilateral amblyopia as VA < 0.30 logMAR (<6/12) in both eyes, with the presence of one or more amblyogenic risk factors and in the absence of ocular pathology that may otherwise explain vision loss. Amblyogenic risk factors included manifest strabismus, anisometropia ≥1.00 D or high refractive error (>5.00 D of hyperopia or myopia). Where available for older children, BCVA obtained following subjective refraction was used to classify amblyopia. In children who were ≤6 years of age for whom BCVA was not available, presenting visual acuity was utilised.

Refractive error was based on the spherical equivalent (SER) refraction in dioptres (D). SER was calculated as sphere + 1/2 cylinder in the eye with reduced vision. Myopia was defined as ≤−0.50 D, hyperopia ≥+2.00 D and astigmatism ≥1.00 D cylinder.

### Statistical Analysis

Data were analysed using IBM SPSS (v22 ibm.com). The distribution of VA (uncorrected, presenting and BCVA) and SER in each age group was calculated by mean (standard deviation (SD)) and median (interquartile range (IQR)). Examination of data normality determined that VA was not normally distributed, while refractive error was normally distributed. However, as reporting of VA is commonly based on the mean, this has been included in addition to the median for comparison. *T*-tests were used to determine whether there was a significant difference in refraction between age groups. For this analysis, data from the right eye only were used due to a high correlation between the right and left eye findings.

The prevalence of reduced vision in each age group was calculated, and chi-square analysis was used to determine if differences in prevalence were statistically significant. Of those who had reduced vision in each age group, the proportion attributed to different ocular conditions was calculated, and chi-square was used again to determine the statistical significance of differences in proportions. A participant was classified as having an ocular condition, including refractive error, when it was present in either or both eyes. The impact of refractive correction on the prevalence of reduced vision was determined by the proportion of reduced vision that was correctable. The incidence of new cases of reduced vision for children aged 12 and 17 years (SAVES) was based on the change in VA from baseline at 6 and 12 years, respectively, with the attributing ocular condition also being determined.

## Results

There was a total of 986 children aged 3–5 years (SPEDS), 1739 children aged 6 years (SMS), 3456 children aged 12 years (SMS *n* = 2345 and SAVES *n* = 1111) and 1649 children aged 17 years (SAVES) who were included in the current analysis. Of the longitudinal cohorts, 978 children were examined at 6 years of age (SMS) and followed up at 12 years (SAVES), while 1362 children were examined at 12 years of age (SMS) and followed up at 17 years (SAVES).

### Normative Visual Acuity and Refraction by Age

**Fig. 1:**
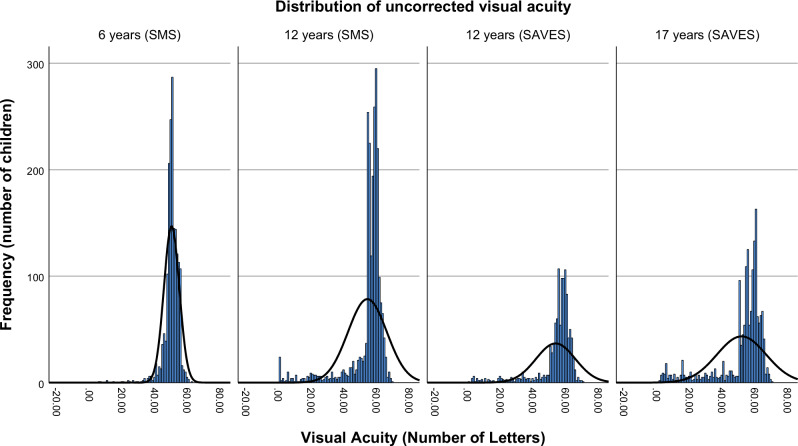
Distribution of uncorrected visual acuity in each cohort. *55 letters is equivalent to 0.00 logMAR (6/6)*. SAVES Sydney Adolescent and Vascular Eye disease Study, SMS Sydney Myopia Study.

Mean uncorrected VA was 0.16 logMAR (6/9.5 + 2) for children aged 3–5 years and 0.10 logMAR (6/7.5) for children aged 6 years (Table 1 and Fig. 1). By 12 years of age, the mean VA had reached close to logMAR 0.00 (6/6) in both the SMS and SAVES cohorts. At 17 years of age, the mean VA was lower at logMAR 0.12 (6/7.5-1), improving to a mean of logMAR −0.04 (6/6 + 2) with refractive correction based on BCVA. When looking at the median uncorrected VA, for children aged 3–5 years this was logMAR 0.0 (IQR:0.1–0.0) (6/6) and logMAR 0.10 (IQR: 0.14–0.06) (6/7.5) (Table 1 and Fig. 1) for children aged 6 years. By 12 and 17 years, the median uncorrected VA had reached ≥ logMAR 0.0 (6/6+).

**Table 1:** Visual acuity and refraction through childhood.

		Presenting VA		Uncorrected VA		Best corrected VA (BCVA)		
Name of study	Age group	Median (Number of. letters read (IQR), LogMAR (IQR)	Mean (Number letters read (Standard deviation), LogMAR	Median (Number of letters read (IQR), LogMAR (IQR)	Mean (Number letters read (Standard deviation), LogMAR	Median (Number of letters read (IQR), LogMAR (IQR)	Mean (Number of letters read (Standard deviation), LogMAR	Mean cycloplegic refraction (SER) (Standard deviation)
SPEDS	3–5 years	55 (IQR:50–55), logMAR 0.00 (IQR: 0.1–0.0)	55 (4), logMAR 0.00	55 (IQR: 50–55), logMAR 0.00 (IQR: 0.1–0.0)	47 (6.5), logMAR 0.16	Not assessed	Not assessed	+1.29 (1.13)
SMS	6 years	50 (IQR: 48–52), logMAR 0.10 (IQR: 0.14–0.06)	50 (4.04), logMAR 0.10	50 (IQR: 48–52), logMAR 0.10 (IQR: 0.14–0.06)	50 (4.7), logMAR 0.10	Not assessed	Not assessed	+1.27 (0.88)
	12 years	57 (IQR: 54–59), logMAR −0.04 (IQR: 0.02– (−0.08))	56 (6.4), logMAR −0.02	57(IQR: 54–59), logMAR −0.04 (IQR: 0.02– (−0.08))	54 (12.9), logMAR 0.02	55 (IQR: 53–58), logMAR 0.00 (IQR: 0.04–(−0.06))	55 (4.9), logMAR 0.00	+0.49 (1.34)
SAVES	12 years	57 (IQR: 53–59), logMAR −0.04 (IQR: 0.04–(−0.08))	55 (8.4), logMAR 0.00	57(IQR: 53–59), logMAR −0.04 (IQR: 0.04–(−0.08))	53 (12.0), logMAR 0.04	55 (IQR: 53–59), logMAR 0.00 (IQR: 0.04–(−0.08))	54 (5.5), logMAR 0.02	+0.47 (1.33)
	17 years	57 (IQR:53–60), logMAR −0.04 (IQR: 0.04–(−0.1))	56 (7.4), logMAR −0.02	56 (IQR: 50–60), logMAR −0.02 (IQR: 0.1–(−0.1))	51 (15.1), logMAR 0.08	55 (IQR: 52–58), logMAR 0.00 (IQR:0.06–(−0.06))	57 (4.0), logMAR −0.04	+0.03 (1.49)

Data from right eye only.

*IQR* interquartile range, *SER* spherical equivalent refraction, *SAVES* Sydney Adolescent and Vascular Eye disease Study, *SMS* Sydney Myopia Study, *SPEDS* Sydney Paediatric Eye Disease Study, *VA* visual acuity.

**Fig. 2:**
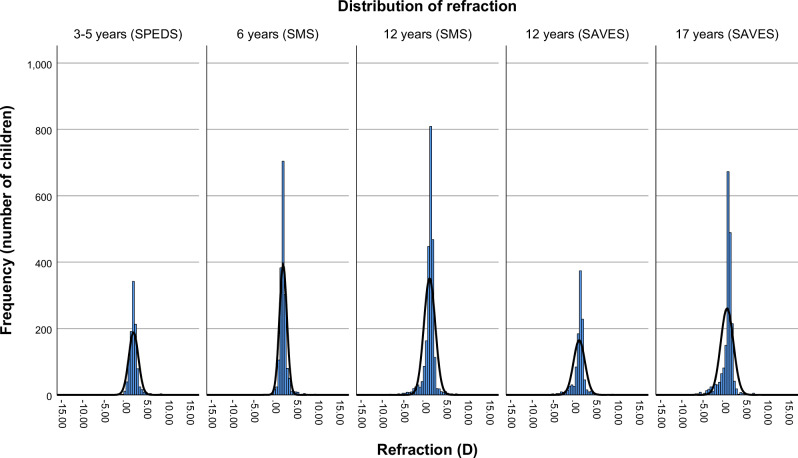
Distribution of refraction in each cohort*.* SAVES Sydney Adolescent and Vascular Eye disease Study, SMS Sydney Myopia Study, SPEDS Sydney Paediatric Eye Disease Study.

The mean SER at 3–5 years of age was +1.29D and remained stable in the 6 year old SMS cohort (+1.27 D), with no significant difference in refraction between these two age groups (*p* = 0.50, Table 1 and Fig. 2). There was a subsequent myopic shift in mean SER by 12 years of age, with a mean of +0.49D and +0.47D in SMS and SAVES, respectively. This further reduced to a mean of +0.03D at 17 years in SAVES (*p* < 0.0001). There was a statistically significant difference in mean SER between the younger (SPEDS 3–5 years and SMS 6 years) and the older age groups at 12 years (SMS and SAVES) and 17 years (all *p* < 0.0001).

The overall prevalence of reduced vision increased with age, with a significant difference between all age groups (*p* < 0.001). The lowest proportion of reduced vision was found in the 3–5 year old children (4.5%) and the highest in the 17 year olds (17.7%) (*p* < 0.001) (Table 2). There was no statistically significant difference in the prevalence of reduced VA between the two 12-year age groups (SMS and SAVES) (*p* = 0.30). The severity of reduced vision also increased with age (*p* < 0.001), with the majority in the younger age cohorts with reduced vision being classified as having only mildly reduced vision (3–5 years: 77.3%, 6 years: 78.9%), compared to the older age groups (12 and 17 years), where most reduced vision was classified as moderate (46.7–69.4%). Additionally, the proportion of those with bilateral reduced vision initially decreased from the 3–5 year age group (52.3%) to 6 years (32.4%), after which it increased with age. The differences in the prevalence of bilateral reduced vision between all age groups were statistically significant (all *p* < 0.001). This includes a significant difference between the two 12-year-old age groups (*p* = 0.02), with a 4% higher proportion of bilateral reduced vision in the SAVES cohort.

**Table 2:** Prevalence of reduced uncorrected visual acuity (<6/12) and severity by age throughout childhood.

			Children with reduced vision			
Age group	Total number of children	Overall prevalence of reduced vision *n (%)*	Mild reduced vision *n (%)*	Moderate reduced vision *n (%)*	Severe reduced vision *n (%)*	Bilateral reduced vision *n (%)*
3–5 years (SPEDS)	986	44 (4.5%)	34 (77.3%)	9 (20.5%)	1 (2.3%)	23 (52.3%)
6 years (SMS)	1739	71 (4.1%)	56 (78.9%)	14 (19.7%)	1 (1.4%)	23 (32.4%)
12 years (SMS)	2345	257 (10.9%)	103 (40.1%)	120 (46.7%)	34 (13.2%)	164 (63.8%)
12 years (SAVES)	1111	134 (12.1%)	35 (26.1%)	93 (69.4%)	6 (4.5%)	91 (67.9%)
17 years (SAVES)	1649	292 (17.7%)	111 (38.0%)	162 (55.5%)	19 (6.5%)	212 (72.9%)

*SAVES* Sydney Adolescent and Vascular Eye disease Study, *SMS* Sydney Myopia Study, *SPEDS* Sydney Paediatric Eye Disease Study.

### Causes of Reduced Uncorrected Vision by Age

#### 3–5 Year Olds

Of the 44 children with reduced vision in the 3–5 year age group, 77.3% and 20.5% had mild and moderate reduced vision, respectively. The primary cause of reduced vision was refractive error (*n* = 30, 68.2%), with the most common being significant astigmatism (*n* = 25) and hyperopia (*n* = 17) (Table 3). Of the hyperopic children, only three had hyperopia alone, and all other children with hyperopia had an additional condition that could account for their reduced vision. In most cases (64.7%), this additional refractive condition was for significant astigmatism. There were an additional 18.1% who had a myopic refraction. The other 14 children with non-refractive causes of reduced vision had conditions such as amblyopia, strabismus and ocular pathology.

**Table 3:** Common causes of reduced uncorrected vision in childhood with age.

Causes of reduced vision	3–5 years (SPEDS) (*n* = 44) *n* (%)	6 years (SMS) (*n* = 71) *n* (%)	12 years (SMS) (*n* = 257) *n* (%)	12 years (SAVES) (*n* = 134) *n* (%)	17 years (SAVES) (*n* = 292) *n* (%)
Amblyopia	9 (20.5%)	20 (28.2%)	38 (14.8%)	5 (3.7%)	0 (0.0%)
Strabismus	4 (9.0%)	18 (25.3%)	28 (10.9%)	11 (8.2%)	10 (3.4%)
Anisometropia	3 (6.8%)	16 (22.5%)	130 (50.6%)	30 (22.4%)	44 (15.1%)
Myopia	8 (18.1%)	13 (18.3%)	215 (83.7%)	111 (82.8%)	240 (82.2%)
Hyperopia	17 (38.6%)	33 (46.5%)	19 (7.4%)	9 (6.7%)	13 (4.5%)
Astigmatism	25 (56.8%)	39 (54.9%)	86 (33.5%)	40 (29.9%)	88 (30.1%)
Ocular pathology	1 (2.3%)	6 (8.5%)	15 (5.8%)	9 (6.7%)	21 (7.2%)

*SAVES* Sydney Adolescent and Vascular Eye disease Study, *SMS* Sydney Myopia Study, *SPEDS* Sydney Paediatric Eye Disease Study.

#### 6 Year Olds

In the 6-year-old SMS group, 4.1% (*n* = 71) were classified as having reduced vision, with 78.9% (*n* = 56) having mild reduced vision, 19.7% (*n* = 14) with moderate reduced vision and 1.4% (*n* = 1) having severely reduced vision (Table 2). The most frequent condition associated with reduced vision was refractive error (88.7%, *n* = 63), including myopia (18.3%), hyperopia (46.5%) and/or significant astigmatism (54.9%). Despite this, only 31 of the 63 children with reduced vision attributed to significant refractive error were found to have spectacles. When testing VA with their refractive correction, 21 were no longer classified as having reduced vision (Table 4). Of the remaining 10 children, only three had an improvement in their vision when wearing their refractive correction, from moderate to mild reduced vision. The other seven children still had the same severity of reduced vision while wearing their refractive correction. Out of these 10 children, 50% had amblyopia, 50% required an update in refractive correction and one child had ocular pathology. Other overall causes of reduced vision in this age group were amblyopia (28.2%) and strabismus (25.3%) (Table 3).

**Table 4:** Reduced uncorrected vision by refractive error, refractive correction use and BCVA outcomes in children with reduced vision.

Age group	Number with refractive error of those with reduced vision	Percentage with refractive correction	Percentage with reduced vision even with refractive correction	Reduced vision after best corrected visual acuity
6 years (SMS)	63	49.2% (*n* = 31)	32.3% (*n* = 10)	–
12 years (SMS)	238	82.8% (*n* = 197)	27.4% (*n* = 54)	2.5% (*n* = 5)
12 years (SAVES)	124	58.1% (*n* = 72)	18.1% (*n* = 13)	0
17 years (SAVES)	265	81.1% (*n* = 215)	4.2% (*n* = 9)	0

*SAVES* Sydney Adolescent and Vascular Eye disease Study, *SMS* Sydney Myopia Study, *BCVA* best-corrected visual acuity.

#### 12 Year Olds

There were two cohorts of 12-year-old children, one from SMS and the other from SAVES (follow-up of 6-year-olds SMS cohort), who were analysed separately. For both 12-year-old cohorts, the proportion of children with reduced vision was just over 10% (SMS 12 years: 10.9% and SAVES 12 years: 12.1%), which was more than double the rate of reduced vision for the children aged 6 years (all *p* < 0.001, Table 2). The majority of 12-year-old children with reduced vision were classified as having moderately reduced vision (SMS 12 years: 46.7% and SAVES: 69.4%). The proportion of those with severely reduced vision was substantially higher in the children aged 12 years in SMS (13.2%) compared to the younger sample aged 6 years (1.4%). In contrast, the rate of severely reduced vision in the SAVES 12-year-old cohort was considerably lower (4.5%) than that of the same age in SMS. There was a greater proportion of children with bilateral reduced vision in both 12-year-old cohorts (SMS: 63.8%, SAVES: 67.9%) than in the younger children aged 6 years.

This corresponded with a high number of children with refractive error in both cohorts (*n* = 362), specifically myopia, which was the attributed cause for a high proportion of reduced vision at 12 years of age (SMS: 83.7%, SAVES: 82.8%, Table 3). Additionally, for the SMS cohort, there was a higher proportion of anisometropia (50.6%), significantly greater (all *p* < 0.001) than other age groups, including the 12-year-old children from SAVES (22.4%). Of those with reduced uncorrected vision with significant refractive error at 12 years, 74.3% (*n* = 269) of both cohorts had habitual refractive corrections, although a quarter still had reduced vision when their VA was tested with their refractive correction in place (Table 4). The prevalence of reduced vision declined with BCVA through subjective refraction, after which, only five children still had reduced vision in the SMS 12-year-old cohort, caused either by residual amblyopia or ocular pathology. No children remained with reduced vision in the SAVES 12-year-old cohort when BCVA was tested. There were 14.8% and 3.7% of children with reduced vision attributed to amblyopia in the SMS and the SAVES cohort at age 12 years, respectively (Table 3).

#### 17 Year Olds

A total of 17.7% (*n* = 292) of children were classified as having reduced vision at 17 years of age, with 38.0% (*n* = 111) classified as mild, 55.5% (*n* = 162) as moderate and 6.5% (*n* = 19) as having severely reduced vision (Table 2). This represents a further increase in reduced vision in this age group compared to those 12 years of age. Again, in the 17-year age group, refractive error (90.8%, *n* = 265) accounted for the largest proportion of reduced vision, specifically myopia (82.2%) (Table 3). Of the children with refractive error, most (*n* = 215, 81.1%) already had a refractive correction; a higher proportion than in 12-year-old children (75%). Only nine of the 215 children with a refractive correction in the 17-year age group had an incorrect or outdated refractive prescription and were classified as having reduced vision even when tested with their refractive correction in place (Table 4). With BCVA, all of these children were classified as no longer having reduced vision. There were also less cases of strabismus and no children with amblyopia in this age group (Table 3).

Across all age groups, ocular pathology accounted for only a very small proportion of reduced vision (Table 3). Identified conditions included inherited retinal dystrophy consistent with Leber congenital amaurosis, Coats disease, ocular albinism and isolated retinal abnormalities. In contrast to refractive error, ocular pathology contributed minimally to the overall reduced vision.

### Incidence of New Cases at Follow-up

Table 5 shows the incidence of new cases of reduced vision in the 5–6 year follow-up of the SMS cohorts, as part of SAVES. Participants included in this analysis comprised 978 children examined at age 6 years and followed-up at 12 years, and 1362 children examined at 12 years and followed-up at 17 years. Between the ages of 6 and 12 years, there were an additional 66 cases of reduced vision (7.2% incidence rate per 6 years), with the predominant cause being myopia and just over 50% already having been prescribed a refractive correction. From 12 years to 17 years of age, there were 79 additional cases (6.4% incidence rate per 5 years) of reduced vision, with the most prevalent condition again being myopia. Almost three-quarters of the children with reduced vision had already been prescribed a refractive correction.

**Table 5:** Incidence of new cases of reduced uncorrected vision at 5–6 year follow-up.

	Incident cases of uncorrected reduced vision, worse than 0.30 logMAR (6/12)	% with refractive correction at follow-up	Predominant cause of reduced vision for incident cases at follow-up
6 year olds followed-up at age 12 years of age	66	51.5%	Myopia – 88%
12 year olds followed-up at age 17 years of age	79	73.4%	Myopia – 66.2%

## Discussion

This paper aimed to identify the prevalence and common causes of reduced vision through childhood and into adolescence, with the intention of understanding the proportion of reduced vision and ocular conditions that may be detected by visual acuity screening at different ages. While visual impairment has been described previously in preschool and school-aged children [10, 37], the prevalence of reduced vision and attributed causes is likely to vary with age and location, as well as over time, given the increases that have been seen in the prevalence of myopia in recent decades [27]. It is important to define the expected proportion of referrals and conditions likely to be detected for each screening population. The availability of population-based data for children from preschool to 17 years of age from the collective Sydney Childhood Eye Disease Studies has allowed a novel analysis comparing reduced vision and attributed causes across ages for Australian urbanised children. The availability of longitudinal data from SAVES uniquely provides a further opportunity to investigate incident cases of reduced vision through the school years and whether repeated screening may be necessary at older ages.

The rate of uncorrected reduced vision stayed consistent at just above 4% among preschool children (ages 3–5 years) and those in the early school age (6 years). However, the prevalence increased progressively with age, reaching a peak of 18% by the end of secondary schooling (17 years). The severity of reduced vision also increased with age. This was in parallel to increases in prevalent myopia with age, which was present for more than 80% of children with reduced vision in the older cohorts. Age-normative mean visual acuity, increased initially for children between 3–5 and 6 years, and 6 and 12 years, at which time a mean of logMAR 0.0 (6/6) was reached, coinciding with the visual system being fully developed [38].

The recommended age for vision screening is preschool age [14, 17, 39–42], corresponding to the current study’s 3–5 year old age group. However, some screening programmes still target the early school years at approximately 5–6 years of age [43–51]. The present examination of data from large population-based studies found that the prevalence of reduced vision was 4.1% and 4.5%, respectively, in these age groups. This suggests that  in terms of finding ‘additional’ ocular conditions at school entry compared to pre-school age, there is no advantage for this Australian urbanised population. It should be noted that a slightly higher cut-off for reduced vision (logMAR 0.30, <6/12) was used here, rather than the typically used referral cut-off (logMAR 0.20, <6/9) for pre-school vision screening programmes [52–54]. Direct comparison with referral rates from vision screening programmes is not practical, as these are likely to be higher than the significantly reduced vision in these populations. The current referral cut-offs used in vision screening programmes represent a conservative approach to referral and shifting to a cut-off of  worse than 6/12 (>0.30 logMAR) would increase the sensitivity but reduce the specificity of a vision screening programme. The lower referral cut-off has been deemed appropriate for a pre-school vision screening programme and is likely to reduce false negative screening [55–57].

The cut-off of <6/18 (logMAR 0.48) used in this study for moderately reduced uncorrected vision corresponds more closely to the criteria for a ‘high priority’ referral (≥6/18) from screening in the New South Wales Statewide Eyesight in Preschooler Screening (StEPS) programme [52]. The current analysis, based on VA testing and a comprehensive diagnostic assessment by orthoptists, estimates that the screening programme should have approximately 1% of the total screened population referred as ‘high priority’ in preschool-aged children. This is somewhat less than the high-priority rate (2.2%) found in the StEPS programme [52]. This difference can be explained, in part, by the variation in cut-off VA used and that the data was collected by experienced orthoptists in the Sydney Childhood Eye Studies, compared with nurses and lay screeners in the StEPS programme.

The most common eye conditions found in the present study indicate that vision screening at preschool age is most likely to find refractive errors, followed proportionally by amblyopia and strabismus. This corresponds with target conditions for childhood vision screening, although the emphasis tends to be given to amblyopia detection due to the need for early treatment [8, 39, 58, 59]. The main goal of vision screening is to detect conditions that are ‘invisible’ or not readily noticed by parents or reported by the child, and as such, they are unlikely to seek the required medical attention. Such ‘invisible’ conditions include amblyopia, as it is most commonly unilateral and may not be noticed due to the other eye having good vision [52]. Correction of refractive errors that reduce visual acuity significantly is beneficial, particularly prior to school entry [52, 60–62] and in cases of early-onset myopia, detection at a young age provides the opportunity to commence optical and/or pharmacological interventions to reduce progression to high myopia [63–67]. Thus, there is considerable value in detecting refractive errors early, and this should be considered a benefit and important aim of early vision screening alongside amblyopia detection. These findings support current recommendations for screening to be conducted at preschool age to support optimal amblyopia treatment outcomes and correction of vision prior to school commencement.

Amblyopia accounted for 21% and 28% of reduced vision, respectively, in the two youngest age cohorts (3–5 years and 6 years). There was a substantial decline in the relative proportion of reduced vision attributed to amblyopia as children aged, whereas other causes, including refractive error and to a lesser extent, ocular pathology, increased in prevalence. Interestingly, the percentage of children with amblyopia was lower in the SAVES 12-year-old cohort than those from SMS at the same age. A possible reason for this is that the SAVES 12-year-old children were a follow-up of the SMS 6-year-olds, and may have been identified as amblyopic at 6 years of age, allowing for treatment of their amblyopia to commence. A further reduction in amblyopia prevalence was observed between 12 and 17 years in the SAVES cohort. While this may partly reflect the effects of treatment, complete resolution of true amblyopia during adolescence would be unlikely in the absence of documented treatment history. Alternative explanations include misclassification at younger ages and potential attrition bias resulting from differential loss to follow-up. The Paediatric Eye Disease Investigator Group (PEDIG) have shown that refractive correction alone can result in significant improvement in visual acuity in children with anisometropic and bilateral refractive amblyopia, with additional gains achieved where occlusion or atropine therapy is required [68, 69]. Although amblyopia treatment can still be undertaken at 6 years of age, success has been reported to be lower, and those with moderate or severe amblyopia may not achieve optimal visual acuity in the affected eye [70]. Therefore, late detection of amblyopia leaves children at an increased risk of vision impairment and blindness in adulthood, in the situation that the better-seeing eye suffers from disease or injury [71].

Strabismus is not typically the focus of vision screening programmes, as visual acuity may not be affected, unless strabismic amblyopia develops or there is a concurrent abnormality. There is also some evidence that constant manifest strabismus may be detected by family observation and care sought prior to preschool vision screening [72, 73]. Nevertheless, children with strabismus and reduced vision due to refractive error or strabismic amblyopia can be identified on vision screening, providing an important pathway into care. In the 3–5 year old age group, strabismus was only present in 9% of children with reduced vision; this increased to 25% in the 6 year old children before a steady decline to a low of 3% in 17 year olds. The increase at 6 years could be related to the onset of intermittent strabismus in particular. As strabismus carries psychosocial implications for children in their social settings, such as at school [9, 32, 74], detection and treatment are likely to have a positive impact. There is also the potential for the prevention of amblyopia and maintenance of vision. Some screening protocols include observation and stereopsis testing to increase the possibility of detecting strabismus that has not impacted visual acuity significantly [52]; however, it is unclear whether there is a significant added benefit, and this may also require additional training for screeners.

Most cases of reduced vision across all age groups were due to uncorrected refractive error, aligned with findings from other studies that indicated refractive error to be the main cause of vision impairment in childhood [4, 41, 75, 76]. In agreement with previous studies on preschool children, astigmatism was a major cause of reduced vision in children aged 3–6 years [77–81]. A number of children with reduced vision at younger ages were also found to have hyperopia. However, it is known that children with hyperopia are able to accommodate, effectively compensating for their refractive error on visual acuity testing [82]. In the small proportion of children with hyperopia and reduced vision, an additional condition was present that could account for their reduced vision. Thus, visual acuity screening alone is unlikely to detect the majority of significant hyperopia in children, but hyperopia may be detected incidentally when present concurrently with another ocular condition that causes reduced vision. Previous research has demonstrated that hyperopia may be missed by VA-only screening approaches [83]. Importantly, uncorrected hyperopia has been associated with reduced educational attainment and decreased efficiency of near visual function in school-aged children [60, 84–86]. Therefore, while hyperopia may not always manifest as reduced distance visual acuity, failure to detect and correct clinically significant hyperopia may have broader functional and academic implications. This limitation should be considered when interpreting the scope of conditions detectable through visual acuity-based screening programmes. This may warrant consideration of alternative or adjunct screening approaches where hyperopia prevalence and educational impact are of concern.

There was an increase in anisometropia as a cause of reduced vision in the 12-year-olds from SMS only, which may be due to uneven axial elongation between the eyes with the onset of myopic refractive error at this age [87]. However, this trend was not observed in the 12-year-old children from SAVES, possibly indicating this was a chance finding for this particular cohort. A high proportion of anisometropia as a cause of reduced vision at this age may have implications for binocularity due to a difference in clarity of the two images, and may be an important condition to target in this age group [88]. Fortunately, while anisometropia is a risk factor for amblyopia [32], children at this age have passed the period of neural plasticity within which amblyopia develops [89, 90]. By 17 years of age, the proportion of anisometropia in those with reduced vision was again lower.

There were increases in the overall prevalence of reduced vision at 12 years of age, and a further increase at 17 years of age. This was related to a higher prevalence of myopia in these age groups, corresponding with the typical onset of school myopia, which is said to have its onset around age 10–12 years [91]. Visual acuity testing is highly sensitive for the detection of even low levels of myopia, since myopic refractive error cannot be masked by accommodation in the same way hyperopia may be [82]. The percentage of severe and bilaterally reduced vision also increased in the two older age groups as the levels of myopia rose with ongoing refractive progression.

There is a viewpoint that repeated screening may be necessary at school age to detect new cases of reduced vision caused by the onset of myopia. Approximately 80% of children aged 12 from SMS and 17 years from SAVES with refractive error and reduced vision already had a refractive correction, with the majority having good vision with their prescribed correction. There was a lower rate of refractive correction use in children who were aged 12 years in the SAVES sample (60%), who were unlikely to be myopic at baseline when 6 years of age. The overall high rate of refractive correction use for those with myopia indicates that symptoms of blur may be reported by children of this age, and appropriate care is sought to address the myopia. The longitudinal analysis of incident cases of reduced vision revealed that these were largely caused by myopia and while 73% of 17-year-olds were already using a refractive correction, this rate was lower in 12-year-olds with incident myopia (52%), suggesting that children of this age have not yet reported their reduced vision. It is important to note that access to public health care is likely to have an impact on rates of correction of refractive errors. In Australia, the federal Medicare programme provides free access to optometry care for all citizens [92], and this is likely to have contributed to the high rate of correction of refractive errors. However, prescription spectacles represent an additional cost, which may be a disincentive for some families. These costs can be covered, at least partially, by private health insurance.

Based on the current findings, if additional school screening were to be recommended, it would be most valuable at approximately age 12 years, which corresponds to the final year of primary schooling and the commencement of secondary schooling in Australia. However, there is no indication from the data that repeated screening in the early primary school years is likely to have any substantial benefit. An alternative and likely more cost-effective approach to detecting new onset myopia in older children is to educate parents and children on eye health, with the aim of increasing prompt reporting of symptoms. Since myopia risk can be profiled based on demographics such as ethnic background and parental myopia, as well as risk factor exposure including competitive schooling, low time outdoors and high near work demand [91], there is further potential for targeted myopia screening for those at risk, rather than whole population screening.

The large sample of population-based data across a wide age range used in this analysis has uniquely allowed examination of the prevalence and causes of reduced vision across childhood. The availability of longitudinal population data from the SAVES study is another significant strength of the current analysis. While these datasets provide valuable population-based insight, some data collection occurred up to 20 years ago, and changes in refractive error prevalence, particularly myopia, may limit the generalisability of these findings to contemporary populations. Changes in lifestyle, educational demands and access to eye care over this period may influence both the prevalence and management of refractive errors and amblyopia today. It is to be noted that longitudinal data were only available for analysis between 6 and 12 years and 12 and 17 years of age, respectively. Crucially, longitudinal analysis of new cases of reduced vision could not be conducted between preschool age and school commencement. An additional limitation was that the study did not consider the impact of previous screening or eye examinations on the prevalence of reduced vision. Use of uncorrected visual acuity should somewhat mitigate this impact within this analysis, as it more closely reflects the proportion of reduced vision in an untreated population. However, the impact of previous detection and treatment for amblyopia cannot be determined based on the current analysis and therefore, the proportion of vision loss caused by amblyopia, particularly at older ages, is likely to be higher in a completely unscreened and untreated population.

This study has described the profile of children with reduced vision and the proportion of ocular conditions that are present throughout childhood and are likely to be detected by population vision screening. The results demonstrate that amblyopia, which is a priority to detect and treat early, is a more common cause of reduced vision at a young age. This is supportive of current recommendations for screening to be conducted at preschool ages to facilitate optimal treatment outcomes [16, 17]. Refractive error is a significant cause of reduced vision at all ages, with early myopia and astigmatism a priority for detection in young children, whereas school myopia becomes more prevalent from the age of 12 years onwards. The majority of children with refractive error at older ages had already been prescribed refractive correction, including those who developed reduced vision during follow-up in this longitudinal analysis. It is likely that community education and/or targeted myopia screening at approximately 12 years of age would be more appropriate than repeated population vision screening during the school years. Understanding the profile of the population to be screened is vital for determining the referral burden likely to result from vision screening, and for examining the success of preschool vision screening programmes.

## Data Availability

No datasets were generated or analysed during the current study.
